# Perioperative sleep deprivation activates the paraventricular thalamic nucleus resulting in persistent postoperative incisional pain in mice

**DOI:** 10.3389/fnana.2022.1074310

**Published:** 2022-12-22

**Authors:** Lei Li, Huijie Zhang, Zhenli Zheng, Nan Ma, Yidan Zhang, Yaping Liu, Jingjing Zhang, Songxue Su, Weidong Zang, Jinping Shao, Jing Cao

**Affiliations:** ^1^Department of Anatomy, School of Basic Medical Sciences, Zhengzhou University, Zhengzhou, Henan, China; ^2^Department of Medical Record Management, The First Affiliated Hospital of Xinxiang Medical University, Xinxiang, China; ^3^Jiangsu Key Laboratory of Neuropsychiatric Diseases and Institute of Neuroscience, Soochow University, Suzhou, China; ^4^Neuroscience Research Institute, Zhengzhou University Academy of Medical Sciences, Zhengzhou, Henan, China

**Keywords:** postsurgical pain, paraventricular thalamic nucleus (PVT), CaMKIIα neurons, perioperative sleep deprivation, stress

## Abstract

**Background:**

The duration of postsurgical pain is closely correlated with perioperative stress. Most patients suffer short-term sleep disorder/deprivation before and/or after surgery, which leads to extended postsurgical pain by an undetermined mechanism. The paraventricular thalamus (PVT) is a critical area that contributes to the regulation of feeding, awakening, and emotional states. However, whether the middle PVT is involved in postoperative pain or the extension of postoperative pain caused by perioperative sleep deprivation has not yet been investigated.

**Methods:**

We established a model of postoperative pain by plantar incision with perioperative rapid eye movement sleep deprivation (REMSD) 6 h/day for 3 consecutive days in mice. The excitability of the CaMKIIα^+^ neurons in the middle PVT (mPVT^CaMKIIα^) was detected by immunofluorescence and fiber photometry. The activation/inhibition of mPVT^CaMKIIα^ neurons was conducted by chemogenetics.

**Results:**

REMSD prolonged the duration of postsurgical pain and increased the excitability of mPVT^CaMKIIα^ neurons. In addition, mPVT^CaMKIIα^ neurons showed increased excitability in response to nociceptive stimuli or painful conditions. However, REMSD did not delay postsurgical pain recovery following the ablation of CaMKIIα neurons in the mPVT. The activation of mPVT^CaMKIIα^ neurons prolonged the duration of postsurgical pain and elicited anxiety-like behaviors. In contrast, inhibition of mPVT^CaMKIIα^ neurons reduced the postsurgical pain after REMSD.

**Conclusion:**

Our data revealed that the CaMKIIα neurons in the mPVT are involved in the extension of the postsurgical pain duration induced by REMSD, and represented a novel potential target to treat postoperative pain induced by REMSD.

## Highlights

- Perioperative REMSD delays postsurgical pain recovery.
- REMSD increases the excitability of mPVT^CaMKIIα^ neurons.
- mPVT^CaMKIIα^ activation prolongs the duration of postsurgical pain.

## 1 Introduction

Persistent postsurgical pain (PPP) is a common clinical disorder reported to occur in 10–50% of patients after various common procedures. Although PPP has extensive individual and social consequences, there has been slow progress in the development of new preventive and therapeutic approaches (Vasilopoulos et al., 2021). Many efforts have been made to improve postoperative pain management, and several potential predictors of postoperative pain and several preoperative and psychological factors have been identified. In addition to surgical factors, preoperative psychosocial factors, including anxiety, depression, or self-reported sleep disturbance-induced stress before or after surgery, have been reported to play a vital role in the development and recovery of postsurgical pain (Kehlet et al., 2006; Cao et al., 2015).

Clinical studies have shown that patients sleep less efficiently before surgery and patients with poor sleep report aggravated pain after surgery (Chouchou et al., 2014; Mahran et al., 2020; Guo et al., 2022). Preclinical observations also suggest that short-term sleep deprivation after plantar incision delays recovery from postoperative pain in rodents (Wang et al., 2015; Li et al., 2019). Despite the increasing number of preclinical and clinical studies on the pathophysiology of postoperative pain, how short-term sleep deprivation affects postoperative pain, its underlying mechanism, and therapeutic approaches remain to be further explored. Recently, emerging evidence has showed that brain regions encoding emotional features of pain, pain relief, or sleep and wakefulness may provide a new insight to improve postoperative pain management (Zhou et al., 2019; Guo et al., 2022; Varallo et al., 2022).

As one of the thalamic midline nuclei, the paraventricular thalamic nucleus (PVT) is mainly composed of glutamatergic neurons and receives projections from inhibitory neurons in the brainstem, before integrating the information for later transmission to the cortex. The PVT differentiate three subregions anterior PVT (aPVT), middle PVT (mPVT), and posterior PVT (pPVT). The PVT has been identified as a crucial signal integration hub for many descending and ascending pathways that modulate a variety of behaviors, including circadian rhythm (Kolaj et al., 2012), fear (Do-Monte et al., 2015), vigilance (Petrovich, 2021), anxiety (Kirouac, 2021; Zhao et al., 2022), depression (Zhao et al., 2021), motivation (Haight et al., 2015; Campus et al., 2019; Kirouac, 2021; Wang et al., 2021), and addictive behaviors (Neumann et al., 2016; Choi and McNally, 2017). The role of the PVT in sleep arousal has been widely confirmed. It has been reported that activation of CaMKIIα neurons in the PVT (PVT^CaMKIIα^) promotes the transition from sleep to wakefulness and accelerates recovery from general anesthesia in mice (Ren et al., 2018; Ao et al., 2021). In contrast, inhibition of PVT neurons promotes the sleep process (Kirouac, 2015; Vertes et al., 2022). Moreover, extended wakefulness has been found to increase the expression of c-Fos in the PVT and significantly improve the excitability of PVT neurons during wakefulness, further suggesting that the PVT is a critical thalamic region in the control of arousal (Ren et al., 2018). Moreover, recent evidence suggests that the PVT participates in the regulation of pain, such as in the acute visceral pain response or in the central processing of chronic pain induced by spared nerve injury (Jurik et al., 2015; Chang et al., 2019; Liang et al., 2020). The anterior, middle, and posterior gradient of the PVT displays distinct function and connectivity patterns and recent studies of foundation PVT using advanced approaches have focused on pPVT region (Barson et al., 2020). However, whether the mPVT is involved in postoperative pain or prolonged postoperative pain caused by perioperative sleep deprivation has not yet been investigated.

Rapid eye movement (REM) sleep is associated with brain development (Mueller et al., 2015; Wolfe and Ralls, 2019), memory (Izawa et al., 2019), and stress (Kim et al., 2020). In addition, our previous study demonstrated that perioperative REM sleep deprivation leads to chronic incisional pain at the behavioral level (Cao et al., 2015). In the current study, we established a mouse model of postoperative pain after plantar incision and subjected the mice to perioperative rapid eye movement sleep deprivation (REMSD) 6 h/day for 3 consecutive days after surgery such that their basal pain perception was unaffected, to examine the role of the mPVT in the related postoperative pain process. Using behavioral tests, immunohistochemistry, fiber photometry, and chemogenetic techniques, we provide the first evidence that perioperative REMSD prolonged the duration of postoperative pain by activating mPVT^CaMKIIα^ neurons, which may be regulated by the cortex and brain stem. Our results suggest that the PVT is a critical thalamic area for sleep and pain, and that inhibition of mPVT^CaMKIIα^ neurons eliminated the effect of REMSD on postoperative pain duration.

## 2 Materials and methods

### 2.1 Animals and surgery

C57BL/6J, *CaMKII*α*-Cre*, and *Ai-14* (Rosa26-LSL-Tdtomato) male mice (purchased from the Beijing HFK Bioscience or Shanghai Model Organisms Center) at 8–12 weeks of age were used for the current study. All mice were maintained under a 12-h light/dark cycle (lights on at 7:00 a.m. and lights off at 7:00 p.m.) at a stable temperature (23–26°C) with *ad libitum* access to water and food. All experimental procedures followed the guidelines of the National Institutes of Health and were approved by the Zhengzhou University Animal Care and Use Committee.

### 2.2 Surgery pain

As previously described, animals were anesthetized with 2% isoflurane, a left hind paw 5-mm longitudinal incision was made through the glabrous plantar skin and fascia after disinfecting with 10% povidone-iodine solution (Wang et al., 2015). Under the fascia, the flexor digitorum brevis is raised and cut with bending forceps. After hemostasis with gentle pressure, the skin was sutured with 5.0 suture. The skin was disinfected with iodophor.

### 2.3 REM sleep deprivation procedure

Rapid eye movement sleep disturbance (REMSD) was conducted based on previous studies with minor modification (Wei et al., 2013; Cao et al., 2015). Briefly, mice were placed on a glass platform of 3 cm in diameter in the middle of a Plexiglas tank filled with water filled to 1 cm below the top surface of the platform. The onset of REM sleep was disturbed by muscle atonia that accompanies REM sleep, during which the body comes into contact with water, awakening the animal. Animals were maintained in the tank for 6 h/day for 3 consecutive days during the daytime (10:00–16:00).

### 2.4 Virus injection and optic fiber

After anesthetizing with 2% isoflurane. The mice were fixed in a stereotactic frame (RWD Life Science, Shenzhen, China), the brain surface above the thalamic midline was exposed with a hand drill, and a glass micropipette connected to a microsyringe was inserted into the target location. Virus was injected with a glass pipette at a rate of 20–30 nL/min. Following injection, the pipette was indwelled for 10 min to avoid spillover. At the end of the procedure, the skin was sutured or the optic fiber was fixed through the dental acrylic. Mice infected with the virus were allowed to recover for 3 weeks before behavioral testing.

For chemogenetic manipulation, the AAV2/9-EF1a-DIO-hM3D(Gq)-mCherry (200 nL, 5 × 10^12^ vg/mL, PT-0042, Brain VTA, Wuhan, China) or the control AAV2/9-EF1a-DIO-mCherry was injected into the PVT (−1.50 mm from the bregma, 0 mm lateral from midline, and −3.0 mm vertical from the cortical surface) of *CaMKII*α*-Cre* mice to specifically activate PVT interneurons. For chemogenetic inhibition of PVT interneurons, AAV2/9-EF1a-DIO-hM4D(Gi)-mCherry (200 nL, 5 × 10^12^ vg/mL, PT-0043, Brain VTA, Wuhan, China) or the control AAV2/9-EF1a-DIO-mCherry was injected into the PVT of *CaMKII*α*-Cre* mice. Following virus injection, 1 mg/kg clozapine-N-oxide (CNO) was injected intraperitoneally to observe pain, anxiety, and depression-like behaviors (Ren et al., 2018; Liang et al., 2020). The CNO was injected in the mice at 10:00 for 3 consecutive days after surgery. The AAV2/8-EF1a-DIO-taCasp3-TEVp-P2A-WPRE-hGH (200 nL, 3 × 10^12^ vg/mL, PT-1230, Brain VTA, Wuhan, China) was injected into the PVT of *CaMKII*α*-Cre* mice to specifically ablate glutamatergic neurons in the PVT.

### 2.5 Fiber photometry

After rAAV-EF1a-DIO-GCaMP6s-WPRE (300 nL, 2 × 10^12^ vg/ml, H10010, OBIO, China) was injected into the PVT of *CaMKII*α*-Cre* mice in the stereotaxic coordinate: −1.50 mm from bregma, 0 mm lateral from midline, and −3.0 mm vertical from the cortical surface, the optic fiber [outer diameter (OD) of 200 μm, numerical aperture (NA) of 0.39, RWD Life Science, Shenzhen, China] was placed at the site (−1.50 mm from bregma, 0 mm lateral from midline, and −2.9 mm vertical from the cortical surface). Each mouse was then allowed to recover for 3 weeks before recording.

A fiber photometry system (R810, RWD Life Science, Shenzhen, China) was used to record the fluorescence signals generated by 470-nm LED light and 410-nm LED light excitation. On the day of the experiment, mice were acclimated for 30 min in the behavioral test room, and basal fluorescence was recorded for 5 min after acclimation. Next, mice were stimulated with von Frey (0.07 g, 1.0 g) and acetone. The ΔF/F was calculated according to (470 nm signal–fitted 410 nm signal)/(fitted 410 nm signal). The formula was as follows: Z score = (x-mean)/std, x = ΔF/F. After recording, histological analysis was used to verify the site of viral transduction and the optic fiber. The mice without correct viral transduction or correct optic fiber site were excluded from the analysis.

### 2.6. Behavioral tests

All mice were acclimatized to the laboratory environment 3 days before behavior testing. Mechanical, thermal, and cold hypersensitivity were determined using paw withdrawal frequencies (PWFs), paw-withdrawal latency (PWL), and acetone score, respectively, at 7:00–10:00 on the 1, 3, 5, and 7 days after surgery. Anxiety-like behaviors were determined using the open field test (OFT) and elevated zero maze (EZM) between 10:00 and 18:00 on the 7 days after surgery, meanwhile, the interval between OFT and EPM is more than 4 h. The behaviors of each mouse were scored by individuals blinded to the treatment or experimental grouping.

#### 2.6.1 Mechanical PWF

The PWFs in response to mechanical stimuli were measured as described previously (Minett et al., 2011). Briefly, the mice were placed in a plastic box with a metallic mesh floor and allowed to adapt to the environment for 30 min. Next, calibrated von Frey filaments (Muromachi Kikai, Tokyo, Japan) 0.07 and 0.4 g were used to stimulate the left hind paw middle plantar of the mice 10 times, at 3-min intervals. Lifting, brisk walking, flicking, and licking were regarded as positive responses. The number of positive responses of 10 stimulations was represented as a percentage withdrawal frequency [(number of paw withdrawal/10 trials) *100].

#### 2.6.2 Thermal PWL

The mice were placed in an individual chamber that could be heated by aiming a light beam through the glass plate. Radiant heat was delivered to each hind paw through the glass plate, stimulating the middle of the plantar surface of the left hind paw (Ugo Basile S.R.L., Milan, Italy). The beam automatically cut off when the mouse lifted its foot, and the time from the start of the beam to the hind paw lifting was regarded as the PWL. Each trial was repeated three times, at a minimum interval of 5 min; the final score was the average of the three times. A maximum shut-off time of 20 s was set to avoid any tissue damage.

#### 2.6.3 Acetone score test

The acetone score test was conducted as described previously, with minor modifications (Vissers and Meert, 2005). Briefly, the mice were enclosed in a plastic box with a metallic mesh floor and allowed to adapt to the environment for 30 min. Next, acetone (50 μl) was ejected to the mid-plantar skin surface of the left hind paw with a syringe. The behavior of the mice was observed within 30 s of the acetone spray and were scored according to the following criteria: 0, no response; 1, slight paw withdrawal or flicking; 2, persistent flicking of the hind paw or stomping; and 3, repeated flicking with licking hind paw. This test was repeated three times at 5-min intervals, and the final score was the average of the three times.

#### 2.6.4 Sucrose preference test

The sucrose preference test was performed as described previously (Wang et al., 2015). Before the experiment, mice were individually placed in two identical bottles (one filled with water and the other containing 1% sucrose), and the two bottles were positioned randomly (left or right) from one trial to the next. On the 7th postoperative day, the consumption of each fluid for 24 h was recorded and the sucrose preference was calculated as: sucrose intake (ml)/total intake (ml).

#### 2.6.5 Open field test

The OFT was performed to evaluate locomotion activity and anxiety-like behavior. The mice were placed in the center of the open field apparatus, consisting of a gray Plexiglas box (40 cm × 40 cm × 50 cm), and recorded on a computer camera for 5 min. The time spent in the central squares and the total distance in the box were recorded and analyzed by the video tracking system of Smart v3.0 software (Panlab Harvard Apparatus, USA). After each test, the open-field arena was cleaned with 75% ethanol.

#### 2.6.6 Elevated plus maze test

The elevated plus-maze (EPM) test was used to assess anxiety-like behavior. The elevated plus-shaped maze (60 cm above the floor) consisted of a central platform (5 cm × 5 cm), two open arms (30 cm × 6 cm), and two closed arms (30 cm × 6 cm × 15 cm). In the testing session, each mouse was placed in the central platform facing one of the open arms and allowed to explore freely for 5 min in a dim room. The total distance and time spent in the open arms were recorded using Smart v3.0 software (Panlab Harvard Apparatus, USA). The maze was cleaned with 70% ethanol after each trial.

### 2.7 Immunohistochemistry assay

Animals were deeply anesthetized with vaporized sevoflurane and perfused with 50 ml saline, followed by 50 ml 4% paraformaldehyde (PFA) at 6:00 pm on the 7th postoperative day. The brains were extracted and soaked in 4% PFA at 4°C overnight, cryoprotected by transferring to a 20% sucrose solution (4°C) until the brains were saturated, and then transferred to a 30% sucrose solution until the brains were saturated at 4°C in the refrigerator. Coronal brain sections (30 μm) were cut using a freezing microtome (CM1950, Leica). The slices undergoing immunohistochemical staining were washed in phosphate buffered saline (PBS) three times (10 min each time) and pre-incubated in PBS containing 5% normal goat serum and 0.5% Triton X-100 for 2 h at room temperature, before incubating with the following primary antibodies at 4°C overnight: rabbit anti-CaMKIIα (1:500, ab134041, Abcam, Masschusetts, USA) and mouse anti-Fos B (1:500, ab11959, Abcam, Masschusetts, USA). Then, the sections were washed in PBS three times (10 min each time) and incubated with a secondary antibody at 37°C for 1 h. The slices were washed in PBS three times (10 min each time), stained with DAPI, and washed a further three times with PBS (10 min each time). Finally, all of the slices were mounted onto glass slides, dried, and covered with Antifade Mounting Medium (Solarbio, S2100, Beijing, China). The images were captured using a Leica DMI4000 fluorescence microscope and equipped with a DFC365FX camera (Leica). For the analysis of c-Fos expression in CaMKIIα neurons of PVT, 20 × images were taken, and the number of neurons expressing c-Fos or CaMKIIα were counted in the PVT from three sections for each mouse. The ratio of c-Fos-positive neurons was calculated as follows: (c-Fos/CaMKIIα) *100.

### 2.8 Enzyme-linked immunosorbent assay

The PVT region was harvested on the 7th postoperative day. The supernatants were collected after centrifugation and stored at −80°C until required for use. The level of glutamate in the PVT was determined using an ELISA kit (Meimian, Jiangsu, China) according to the manufacturer’s instructions.

### 2.9 Statistical analysis

All data were collected randomly and presented as mean ± SEM. The data were statistically analyzed with two-tailed *t*-test for comparisons between two groups, or one- or two-way ANOVA with repeated measures for multiple comparisons. Values of *P* < 0.05 were considered statistically significant.

## 3 Results

### 3.1 REMSD prolongs postsurgical pain duration

We defined the number of daily repetitions of REMSD that did not affect the basal pain threshold. We found that exposure to rapid eye movement sleep deprivation 6 h daily for 3 consecutive days did not alter the basal responses to mechanical, heat, and acetone stimuli, but REMSD 6 h/d for 5 consecutive days increased basal pain perception at 7 days after REMSD (Supplementary Figure 1). Thus, REMSD 6 h/day for 3 consecutive days was used in the following experiments.

**FIGURE 1:**
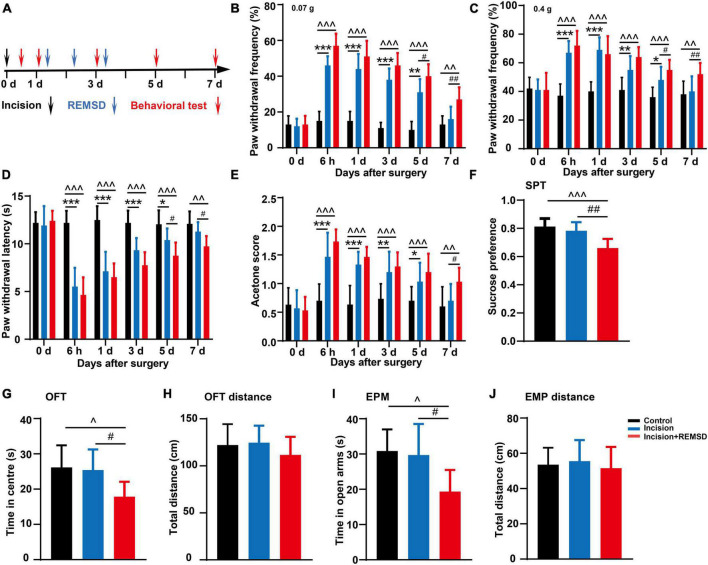
Effect of incision and perioperative rapid eye movement sleep deprivation (REMSD) on postsurgical pain in mice. **(A)** Schematic diagram of the experimental schedule. **(B–D)** Mechanical paw withdrawal frequency in response to 0.07 g von Frey **(B)**, 0.4 g von Frey **(C)**, and paw withdrawal latency in response to thermal stimulus **(D)** at different time points. **(E)** Measurements of cold sensitivity by acetone testing. **(F–J)** The anxiety-like behavioral tests on the 7 days after surgery. **(F)** Sucrose preference test (SPT). **(G,H)** Open field test (OFT) [**(G)** time spent in the center zone; **(H)** total distance in the box]. **(I,J)** Elevated plus maze (EPM) [**(I)** time spent in the open arms; **(J)** total distance of the maze]. Significances of the corresponding time points were assessed by one-way ANOVA with *post hoc* multiple comparisons test in panels **(B–E)**, and ordinary one-way ANOVA *post hoc* multiple comparisons test between groups in panels **(F–J)**. Control vs. incision, *P < 0.05, **P < 0.01, ***P < 0.001. Control vs. incision + REMSD, ^∧^*P* < 0.05, ^∧∧^*P* < 0.01, ^∧∧∧^*P* < 0.001. Incision vs. incision + REMSD, ^#^*P* < 0.05, ^##^*P* < 0.01. *n* = 8 in each group.

To model the effect of perioperative REMSD on postoperative pain, we established a plantar incision-induced postoperative pain model and initiated a REMSD procedure postoperatively 6 h/day for 3 consecutive days. A schematic diagram of the experimental schedule is shown in Figure 1A. Simultaneously, compared to the control group, the mechanical PWFs (0.07 g von Frey and 0.4 g von Frey) and acetone scores in the incision group increased significantly at 6 h, 1, 3, and 5 days, following incision but showed no significant difference on 7 days post-surgery (Figures 1B, C, E). Meanwhile, the thermal PWLs of the incision group decreased significantly on 6 h, 1, 3, and 5 days post-surgery, but recovered on day 7 post-surgery (*P* = 0.429) when compared to the control group (Figure 1D). These data indicate that the incision-induced pain hypersensitivity lasted for 5 days and completely disappeared on day 7 post-surgery. In addition, the mechanical PWFs (0.07 g von Frey and 0.4g von Frey) and acetone scores in the incision + REMSD group increased significantly on 6 h, 1, 3, 5, and 7 days post-surgery (Figures 1B, C, E), as compared to the control group in the corresponding time points. The incision + REMSD group had significantly shorter thermal PWLs on 6 h, 1, 3, 5, and 7 days post-surgery than the control group (Figure 1D). These results showed that pain hypersensitivity of the incision + REMSD group reached a peak at 6 h, and lasted for 1–7 days post-surgery.

In addition, there were no significant differences between the incision + REMSD group and the incision group in the mechanical PWFs and thermal PWLs on the 6 h, 1 and 3 days after surgery and acetone scores on the 6 h, 1, 3, and 5 days after surgery. However, the incision + REMSD group showed significantly higher mechanical PWFs (0.07 g von Frey and 0.4 g von Frey) on days 5 (*P* = 0.005 and *P* = 0.037) and 7 (*P* = 0.008 and *P* = 0.009) after surgery and acetone scores (*P* = 0.031) on days 7 after surgery, as compared to the incision group (Figures 1B, C, E). Moreover, the thermal PWLs of the incision + REMSD group kept significantly lower than the incision group on days 5 (*P* = 0.034) and 7 (*P* = 0.048) after surgery (Figure 1D). Our results indicate that REMSD delays postsurgical pain recovery. Additionally, considering that perioperative sleep deprivation may affect mood or emotional condition, anxiety-like behaviors were also examined. Compared to the incision groups, anxiety-like behaviors were developed in the incision + REMSD group on days 7 after surgery, as evidenced by the OFT (time spent on the center zone declined from 25.4 ± 5.8 s to 17.8 ± 4.2 s, Figure 1G), EPM (time spent on the open arms decreased from 29.7 ± 8.8 s to 19.4 ± 6.1 s, Figure 1I), and SPT (sucrose consumption reduced from 78% ± 6.1% to 66% ± 6.5%, Figure 1F). The mice in the incision + REMSD group showed significantly less sucrose consumption than those in the control group (*P* < 0.001) on days 7 after surgery. In addition, the mice in the incision + REMSD group spent less time on the center zone (*P* = 0.03) and the open arms (*P* = 0.019) than the mice in the control group on the 7 days post-surgery. However, no significant differences were observed in the total distance in the OFT and EPM among the three groups (Figures 1H, J). There were no significant differences between the incision group and the control group in the SPT, OFT, and EPM on the 7 days after surgery. To conclude, perioperative REMSD prolonged the duration of postsurgical pain and evoked anxiety-like behaviors.

### 3.2 REMSD increases the neuronal excitability of mPVT^CaMKIIα^

**FIGURE 2:**
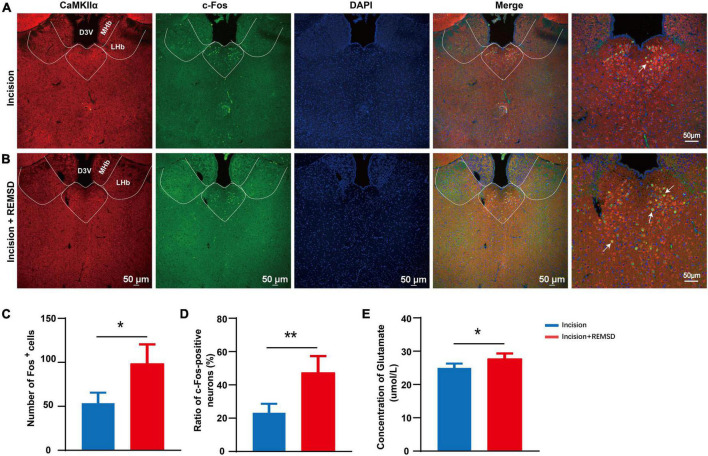
Rapid eye movement sleep deprivation (REMSD) can significantly increase c-Fos in the CaMKIIα^+^ neurons in the middle paraventricular thalamus (mPVT^CaMKIIα^) at day 7 after surgery. **(A)** Expression of c-Fos in the mPVT^CaMKIIα^ of the incision group. **(B)** Expression of c-Fos in the mPVT^CaMKIIα^ of the incision + REMSD group. The arrows represent CaMKIIα neurons colocalize with c-Fos. **(C)** Quantification of c-Fos expressing neurons in incision and incision + REMSD mice and the counted area of c-Fos^+^ neurons was delineated of mPVT by the broken line. **(D)** Ratio of c-Fos-positive neurons in the paraventricular thalamus (PVT) after REMSD treatment. The ratio of c-Fos-positive neurons was calculated as follows: (c-Fos/CaMKIIα) *100. **(E)** Level of glutamate in the PVT after REMSD treatment. Significance was assessed by two-tailed unpaired *t*-test in panels **(C–E)**. Incision + REMSD vs. incision; *n* = 5. *P < 0.05, **P < 0.01.

The PVT has been reported to play an important role in sleep and wake processes, and its role in pain regulation has received increasing attention in recent years. To understand whether the mPVT is involved in the mechanism of REMSD in prolonging the postsurgical pain duration, we assessed the neuronal excitability of mPVT^CaMKIIα^ neurons by examining the expression of c-Fos (Figures 2A, B). Quantification data showed that REMSD increased the number of c-Fos^+^ neurons in the mPVT (incision: 53.5 ± 13.5, incision + REMSD: 98.3 ± 22.2) (Figure 2C). In addition, the positive expression of c-Fos (47.5 ± 10.0%) in CaMKIIα neurons increased remarkably in the incision + REMSD group compared to the incision-only group (23.5 ± 5.4%) on the 7th postoperative day (Figure 2D, *t* = 4.213, *P* = 0.006). Furthermore, compared to the incision group, the concentration of glutamate in the PVT region increased in the incision + REMSD group (Figure 2E, *t* = 2.612, *P* = 0.04). These results indicate that the excitability of mPVT^CaMKIIα^ neurons increased in the prolonged postoperative pain induced by REMSD.

### 3.3 Nociceptive stimulation increases the excitability of mPVT^CaMKIIα^ neurons

**FIGURE 3:**
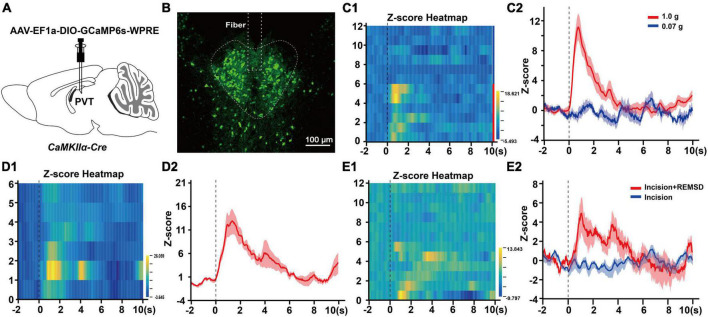
Noxious stimuli increase mPVT^CaMKIIα^ neuronal excitability. **(A)** Experimental configuration showing AAV-EF1a-DIO-GCaMP6s-WPRE injection into the middle paraventricular thalamus (mPVT) of *CaMKII*α*-Cre* mice, and implantation of an optic fiber. **(B)** The injection site of the virus and the location of the optic fiber in the paraventricular thalamus (PVT). Scale bar: 100 μm. **(C1)** Heatmap illustrating the Ca^2+^ transients signals of the mPVT^CaMKIIα^ neurons in the normal mice receiving von Frey filament stimulation. Each row of the heat map stands for one trial of one mouse, the Y-axis stands for the number of tested mice. Mouse 1 to 6 received the 1.0 g von Frey filament stimulation and mouse 7–12 received the 0.07 g von Frey filament stimulation. **(C2)** The average Ca^2+^ transients of mice shown in panel **(C1)**. **(D1)** Heatmap illustrating the Ca^2+^ transients signals of the mPVT^CaMKIIα^ neurons in the normal mice received acetone stimulation. **(D2)** The average Ca^2+^ transients of mice in panel **(D1)**. **(E1)** Heatmap illustrating the Ca^2+^ transients signals of the mPVT^CaMKIIα^ neurons in the mice received 0.07 g von Frey filament stimulation on the 7th day after surgery. The row 1–6 from the incision + REMSD group and the row 7–12 from the incision group. **(E2)** The average Ca^2+^ transients of the incision and incision + REMSD group mice receiving 0.07 g von Frey filament stimulation.

To assess the role of mPVT^CaMKIIα^ neurons in pain modulation, we applied fiber photometry recording *in vivo* to investigate the spontaneous activity of the mPVT^CaMKIIα^ neurons corresponding to external nociceptive stimulation such as mechanical and cold stimulus. Firstly, we injected rAAV-EF1a-DIO-GCaMP6s-WPRE into the mPVT of *CaMKII*α*-Cre* mice and implanted. an optic fiber, then recorded the excitability of mPVT^CaMKIIα^ neurons by fiber photometry (Figures 3A, B). The results showed that the neuronal activity was significantly enhanced by mechanical stimulation with 1.0 g von Frey (noxious stimulus, Figures 3C1, C2) or cold stimulation with acetone spray (Figures 3D1, D2) in normal mice, while mechanical stimulation with 0.07 g von Frey had no significant effect (Figures 3C1, C2). However, the activity of mPVT^CaMKIIα^ neurons increased in response to mechanical stimulation with 0.07 g von Frey in mice in the incision + REMSD group vs. the incision group on day 7 after surgery (Figures 3E1, E2), which was consistent with the obvious mechanical hypersensitivity exhibited by the incision + REMSD group following 0.07 g von Frey stimulation. Taken together, our fiber photometry results indicate that the excitability of mPVT^CaMKIIα^ neurons increased in response to nociceptive stimuli or painful conditions.

### 3.4 Ablation of mPVT^CaMKIIα^ neurons reduces the duration of postoperative pain induced by REMSD

**FIGURE 4:**
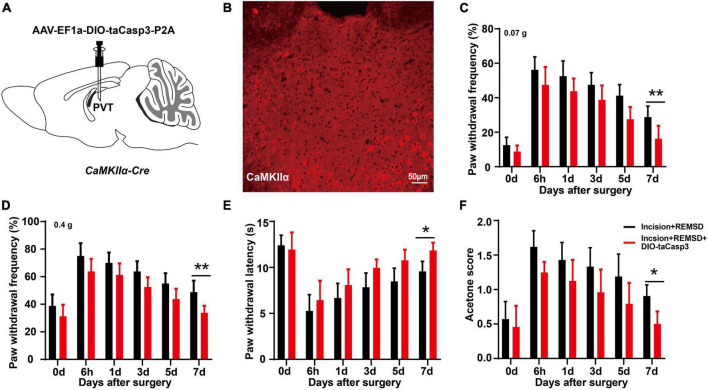
Ablation of PVT^CaMKIIα^ eliminates the prolonged postsurgical pain induced by rapid eye movement sleep deprivation (REMSD). **(A)** Experimental configuration showing AAV-EF1a-DIO-taCasp3-P2A injection into the mPVT of *CaMKII*α*-Cre* mice. **(B)** Image showing CaMKIIα-positive neurons in the middle paraventricular thalamus (mPVT) at 21 days after taCasp3 administration. **(C,D)** Mechanical paw withdrawal frequency in response to 0.07 g von Frey **(C)** and 0.4 g von Frey **(D)** at different time points. **(E)** Paw withdrawal latency in response to thermal stimulus at different time points. **(F)** Measurements of cold sensitivity by acetone testing. Significance was assessed by two-way ANOVA with *post hoc* multiple comparisons between groups in panels **(C–F)**. Incision + REMSD vs. incision + REMSD + DIO-taCasp3; *n* = 8. *P < 0.05, **P < 0.01, scale bar: 50 μm.

Next, we injected AAV-EF1a-DIO-taCasp3-TEVp-P2A into the mPVT of *CaMKII*α*-Cre* mice to specifically ablate the CaMKIIα neurons to further investigate the effect of mPVT^CaMKIIα^ on the prolonged postsurgical pain induced by REMSD (Figure 4A). Moreover, the plantar incision model and REMSD procedure were performed 21 days after the virus injection. The ablation of CaMKIIα neurons was verified by immunofluorescence assay. The results showed that the number of CaMKIIα positive neurons in the mPVT was significantly reduced (from 237 ± 28 to 50 ± 22) after taCasp3 treatment (Figure 4B). Additionally, the function of CaMKIIα neurons were verified by behavior testing, which demonstrated that REMSD did not prolong the duration of postsurgical pain following ablation of CaMKIIα neurons in the mPVT (Figures 4C–F).

### 3.5 mPVT^CaMKIIα^ neuronal activation prolongs the duration of postsurgical pain

**FIGURE 5:**
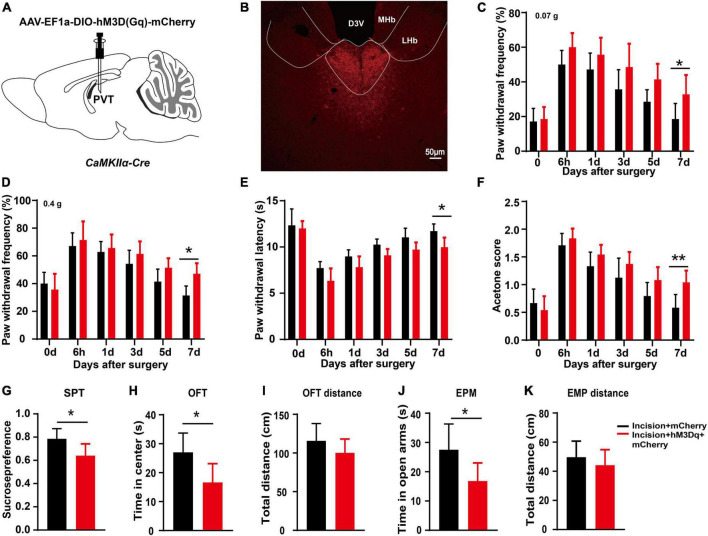
Chemogenetic activation of middle paraventricular thalamus (mPVT)^CaMKIIα^ extended the duration of postsurgical pain. **(A)** Experimental configuration showing AAV-EF1a-DIO-hM3D(Gq)-mCherry injection into the mPVT of *CaMKII*α*-Cre* mice. **(B)** Representative images of viral expression in the mPVT. **(C,D)** Mechanical paw withdrawal frequency in response to 0.07 g von Frey **(C)** and 0.4 g von Frey **(D)** at different time points. **(E)** Paw withdrawal latency in response to thermal stimulus at different time points. **(F)** Measurements of cold sensitivity by acetone testing. **(G)** Sucrose preference test. **(H,I)** Open field test [**(H)** time spent in the center zone; **(I)** total distance in the box]. **(J,K)** Elevated plus maze [**(J)** time spent in the open arms, **(K)** total distance of the maze]. Significance was assessed by two-way ANOVA with *post hoc* multiple comparisons between groups in panels **(C–F)**, two-tailed unpaired *t*-test in panels **(G–J)**. Incision + mCherry vs. incision + hM3D(Gq) + mCherry; *n* = 8. *P < 0.05, **P < 0.01. Scale bar: 50 μm.

To further clarify the effect of mPVT^CaMKIIα^ neurons on the prolonged postsurgical pain induced by sleep deprivation, chemogenetic activation of the mPVT^CaMKIIα^ neurons was achieved by injection of AAV-EF1a-DIO-hM3D(Gq)-mCherry into the mPVT of *CaMKII*α*-Cre* mice (Figures 5A, B). The plantar incision model was established 21 days after virus injection, and CNO was injected intraperitoneally for 3 consecutive days after surgery. We performed behavioral measures to assess the effect of mPVT^CaMKIIα^ neuronal activation on pain and pain-related mood disorders. The chemogenetic activation of the mPVT^CaMKIIα^ neurons by hM3D(Gq) still showed pain hypersensitivity on the 7 days after surgery, demonstrating that the activation of the mPVT^CaMKIIα^ neurons prolonged the duration of postsurgical pain, which was similar to the effect of REMSD (Figures 5C–F). Furthermore, the activation of the mPVT^CaMKIIα^ neurons by hM3D(Gq) elicited anxiety-like behaviors in plantar incision mice, compared to the incision + mCherry group, as evidenced by the SPT (Figure 5G, *t* = 2.631, *P* = 0.025), OFT (Figure 5H, *t* = 2.726, *P* = 0.021), and EPM (Figure 5J, *t* = 2.434, *P* = 0.035). Additionally, no significant differences were observed in the total distance in the OFT and EPM in both the incision + hM3D(Gq) + mCherry group and incision + mCherry group (Figures 5I, K). Collectively, our results showed that similar to the effect of REMSD, the activation of mPVT^CaMKIIα^ neurons prolonged the postsurgical pain duration and elicited anxiety-like behaviors, further supporting the involvement of mPVT^CaMKIIα^ neurons in the prolonged postsurgical pain induced by REMSD.

### 3.6 Inhibition of mPVT^CaMKIIα^ activity eliminates the prolongation of postoperative pain induced by REMSD

**FIGURE 6:**
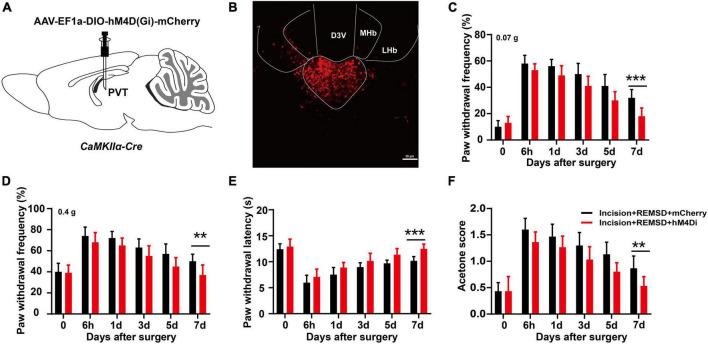
Chemogenetic inhibition of middle paraventricular thalamus (mPVT)^CaMKIIα^ shortened the duration of postsurgical pain induced by rapid eye movement sleep deprivation (REMSD). **(A)** Experimental configuration showing AAV-EF1a-DIO-hM4D(Gi)-mCherry injection into the mPVT. **(B)** Representative images of viral expression in the mPVT. **(C,D)** Mechanical paw withdrawal frequency in response to 0.07 g von Frey **(C)** and 0.4 g von Frey **(D)** at different time points. **(E)** Paw withdrawal latency in response to thermal stimulus at different time points. **(F)** Measurements of cold sensitivity by acetone testing. Significance was assessed by two-way ANOVA with *post hoc* multiple comparisons between groups in panels **(C–F)**. Incision + REMSD + mCherry vs. incision + REMSD + hM4D(Gi) + mCherry; *n* = 10. **P < 0.01, ***P < 0.001, scale bar: 50 μm.

To further determine the role of mPVT^CaMKIIα^ on the prolonged postsurgical pain induced by REMSD. AAV-EF1a-DIO-hM4D(Gi)-mCherry was injected into the mPVT of *CaMKII*α*-Cre* mice to selectively inhibit the CaMKIIα neurons (Figures 6A, B). Twenty-one days later, an incision was made in the plantar aspect of the left hindpaw of the mice followed by REMSD and CNO intraperitoneal injection for 3 consecutive days after surgery. On the 7th day, the mechanical threshold (Figures 6C, D) and thermal paw withdrawal latencies [Figure 6E, *F*_(1,108)_ = 40.52, *P* < 0.0001] distinctly increased in the incision + REMSD + hM4D(Gi) group, while the acetone scores decreased significantly [Figure 6F, *F*_(1,108)_ = 32.64, *P* < 0.0001], compared to the incision + REMSD + mCherry group. These data suggest that REMSD did not delay postsurgical pain recovery when CaMKIIα neurons in the mPVT were inhibited.

## 4 Discussion

Generally, postsurgical pain disappears along with the recovery of the surgical wound. However, approximately 10% of patients still present with moderate to severe chronic pain after wound recovery, which markedly influences the quality of life (Gan et al., 2014). Many of the available analgesics have limited effectiveness or serious side effects, such as nausea/emesis, addiction, tolerance, or hyperalgesia. Therefore, investigating the perioperative risk factors and mechanisms that prolong postsurgical pain is conducive to its intervention and treatment. Most patients undergoing elective surgery can exhibit certain types of stress (e.g., tension, anxiety, and sleep disorder) during the perioperative period, all of which, but particularly sleep disorder, can affect the recovery of postoperative pain. We employed REMSD as the major perioperative stress (6 h/day for consecutive 3 days) without changing the basal pain threshold. We found that such perioperative REMSD prolonged the mechanical, heat, and cold hypersensitivity induced by the incision and elicited short-term anxiety-like behaviors (Figure 1). However, the central mechanisms remain elusive, while a clear understanding of the mechanisms can facilitate recovery from postoperative pain, improvement of the quality of life, and reduction of economic burden in patients.

Sleep deprivation can directly or indirectly impact multiple brain regions involved in pain transmission, among which, the PVT has received much attention due to its role in wakefulness. As a midline thalamus nucleus, the PVT is primarily composed of glutamatergic neurons, mainly vGluT2 neurons and a few vGluT3 neurons, with limited vGluT1 neurons, illustrating that the PVT is mainly composed of excitatory neurons (Ren et al., 2018). It has been reported that the excitation of PVT neurons is closely related to wakefulness. For instance, Hua et al. (2018) found that hunger (aversion) promoted wakefulness *via* the calretinin-positive neurons in PVT. In the current experiment, chemogenetic activation of the mPVT^CaMKIIα^ neurons prolongs incision-induced pain hypersensitivity (Figure 5). Ao et al. (2021) reported that activation of the PVT shortened the awakening time from isoflurane anesthesia in mice. We also found that REMSD led to upregulated expression of c-Fos and level of glutamate in mPVT^CaMKIIα^ (Figure 2). To conclude, PVT activation can accelerate the sleep-to-wake transition.

Recently, the PVT has received increasing attention for its role in pain. Jurik et al. (2015) reported that visceral pain elevated c-Fos expression in the PVT, while pharmacogenetic inhibition of the PVT alleviated visceral pain *via* the descending pain inhibition system. Moreover, a previous study found that the spinal nerve ligation increased the excitability of aPVT neurons to generate pain (Zhang et al., 2022). In the present experiment, fiber photometry was adopted to record the variation of CaMKIIα neuronal excitability. The results showed that non-nociceptive stimulation (0.07 g von Frey) did not lead to an increase in its excitability in normal mice, while both 1.0 g von Frey and acetone stimulation to the hindpaw of mice elevated the excitability of mPVT^CaMKIIα^ neurons (Figures 3C2, D2). These results suggested that external nociceptive stimulation could increase the excitability of PVT^CaMKIIα^ neurons, consistent with the study of Penzo and Gao (2021), where plantar shock was reported to promote PVT neuronal excitability (Ma et al., 2021). Furthermore, on the 7th day after surgery, 0.07 g von Frey did not increase the mPVT^CaMKIIα^ excitability (incision group) but elevated the mPVT^CaMKIIα^ excitability in mice of incision + REMSD group (Figures 3E1, E2), suggesting that REMSD is a cause of increased mPVT excitability after surgery. Collectively, changes in wakefulness and pain states can lead to alteration in PVT neuronal excitability.

To validate the effect of mPVT neurons on the duration of postsurgical pain, we injected taCasp3 virus into the mPVT of *CaMKII*α*-Cre* mice to specifically kill the glutamatergic neurons and found no significant effect on the duration of postsurgical pain in mice undergoing plantar incision plus REMSD. This finding proved the crucial role of glutamatergic neurons in postsurgical pain-induced REMSD. Subsequently, we found that chemogenetic activation of the mPVT neurons prolonged the duration of postsurgical pain (Figure 5), and that inhibition of glutamatergic neuron excitability could shorten the duration of postsurgical pain due to REMSD. This is in agreement with the findings of Antti Pertovaara et al., who showed that metabotropic glutamate receptor 5 (MGluR5) or NMDA receptor antagonist could alleviate the mechanical hypersensitivity induced by rapid eye movement sleep deprivation (Ansah et al., 2010). It has been reported that perioperative factors that lead to sleep disturbance and prolonged waking hours can extend the duration of postsurgical pain, and may cause progression from acute to intractable chronic pain. In case of decreasing the excitability of PVT neurons and improving the patient’s sleep quality, the postsurgical pain may be alleviated, shedding light on the treatment of postsurgical pain accompanied by sleep disorders.

The PVT serves as a relay station for ascending and descending information transmission and can be activated by perioperative stress (Penzo and Gao, 2021). Classical anatomical studies show that the PVT extends over the entire rostrocaudal length of the midline thalamus and displays unique efferent and afferent connectivity patterns with the cortex, basal forebrain, amygdala, ventral striatum, hippocampus, hypothalamus, and brainstem (Vertes and Hoover, 2008; Li and Kirouac, 2012). The above mention brain regions are tightly correlated with pain and negative emotions. A recent study using calcium imaging and c-Fos staining showed that negative emotions activated PVT neurons and simultaneously activated PBN-PVT projections to induce depression and anxiety behaviors (Zhu et al., 2022). This suggests that the PVT primarily regulates negative emotions by receiving signals from the brain stem. In addition, the PVT regulates negative emotions by integrating the mPFC cognitive signals and internal state signals of the hypothalamus and brainstem (Christoffel et al., 2021; Dumont et al., 2022). In other words, the PVT integrates corresponding signal inputs and play the role of descending facilitatory system. Based on the close relationship between the PVT, sleep, and pain, we believe that the activation of the PVT is a significant factor involved in the extension of postsurgical pain induced by perioperative REMSD.

To summarize, we adopted multiple methods (chemogenetics, fiber photometry, and molecular techniques) to prove that mPVT activation prolonged the duration of postsurgical pain, and clarified that the mPVT participated in the regulation of negative emotions. In the experiment, our study is centered on the function of the mPVT in postsurgical pain induced by REMSD. However, whether or not the anterior/posterior of the PVT affects the recovery of postsurgical pain is still largely unknown. In addition, which genotypic differences contribute to the heterogenous activity in postsurgical pain remains unclear. In following experiments including high-throughput single-cell sequencing or targeting of unique cell types would focus on elucidating how these genetically distinct cell populations contribute to pain behavior.

As the PVT is the key region that integrates and transmits ascending and descending signals, its downstream functions remain unexplored. In the future, we will continue to take the PVT as a key research direction to explore the upstream and downstream regulatory and regulated brain regions, to elucidate the network relationship between sleep and pain to offer personalized prevention and treatment for patients with postsurgical pain.

## Data availability statement

The original contributions presented in this study are included in the article/Supplementary material, further inquiries can be directed to the corresponding authors.

## Ethics statement

This animal study was reviewed and approved by Zhengzhou University Animal Care and Use Committee.

## Author contributions

LL, JS, WZ, and JC: study conception and design. LL, HZ, ZZ, NM, YZ, YL, and JC: data acquisition and analysis. LL, JZ, and JC: drafting figures. LL, SS, JS, and JC: drafting manuscript. JC: finalizing manuscript. All authors contributed to the article and approved the submitted version.
