# Supplementary material for: Perioperative sleep deprivation activates the paraventricular thalamic nucleus resulting in persistent postoperative incisional pain in mice
Source: Front Neuroanat. 2022 Dec 22;16:1074310. doi: 10.3389/fnana.2022.1074310 (PMC9813598; doi:10.3389/fnana.2022.1074310)
Supplement: Supplementary file 1 [file Data_Sheet_1.pdf]

Supportive information:

The mice were divided into a control group, a rapid eye movement sleep deprivation (REMSD) 3 d group (REMSD + 3 d), and the REMSD 5 d group (REMSD + 5 d). The mice of the REMSD + 3 d group and REMSD +5 d group were exposed to REMSD 6 h daily for 3 or 5 days, respectively. The REMSD + 3 d group did not alter the basal responses to mechanical, heat, and acetone stimuli when compared to the control group. However, significant decreases in paw withdrawal thresholds in response to mechanical stimulation, heat, and cold were observed only in the REMSD + 5 d group on the 7 days after sleep deprivation as compared to the corresponding control group.

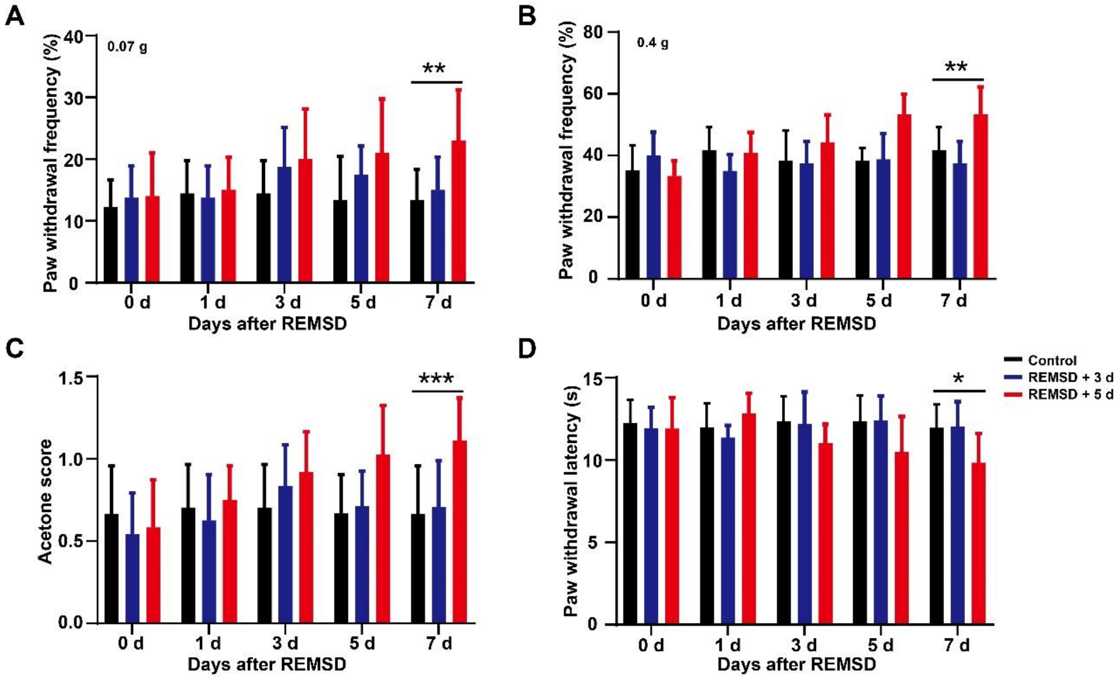

Fig 1. The changes in basal paw withdrawal responses in mice to mechanical, heat, and cold stimuli after rapid eye movement sleep deprivation (REMSD) 6 h daily for 3 and 5 consecutive days. Mechanical paw withdrawal frequency in response to 0.07 g von Frey (A) and 0.4 g von Frey (B) at different time points. C. Paw withdrawal latency in response to the thermal stimulus at different time points. D. Measurements of cold sensitivity by acetone testing. Significance was assessed by two-way ANOVA with post hoc multiple comparisons between groups in A-D. Control *vs* REMSD + 5 d, * P <0.05, ** P <0.01, *** P <0.001.
